# *Campylobacter coli* infection causes spinal epidural abscess with Guillain–Barré syndrome: a case report

**DOI:** 10.1186/s12883-021-02537-6

**Published:** 2022-01-03

**Authors:** Masako Fujita, Tatsuya Ueno, Michiru Horiuchi, Tatsuro Mitsuhashi, Shouji Yamamoto, Akira Arai, Masahiko Tomiyama

**Affiliations:** 1 Department of Neurology, Aomori Prefectural Central Hospital, 2-1-1 Higashi-Tsukurimichi, Aomori, 030-8551 Japan; 2 Department of Infection Control Office, Aomori Prefectural Central Hospital, Aomori, Japan; 3 Department of Bacteriology I, National Institute of Infectious Diseases, Tokyo, Japan; 4 Department of Neurology, Hirosaki University Graduate School of Medicine, Hirosaki, Japan

**Keywords:** *Campylobacter coli*, Guillain–Barré syndrome, Spinal epidural abscess, Bacterial infection; acute motor sensory axonal neuropathy; paralysis, Anti-ganglioside antibodies

## Abstract

**Background:**

Guillain–Barré syndrome (GBS) and spinal epidural abscess (SEA) are known as mimics of each other because they present with flaccid paralysis following an infection; however, they differ in the main causative bacteria. Nevertheless, the two diseases can occur simultaneously if there is a preceding *Campylobacter* infection. Here, we report the first case of SEA with GBS following *Campylobacter coli* infection.

**Case presentation:**

A 71-year-old Japanese man presented with progressive back pain and paralysis of the lower limbs following enteritis. Magnetic resonance imaging showed a lumbar epidural abscess that required surgical decompression; therefore, surgical drainage was performed. Blood cultures revealed the presence of *C. coli*. Despite surgery, the paralysis progressed to the extremities. Nerve conduction studies led to the diagnosis of GBS. Anti-ganglioside antibodies in the patient suggested that GBS was preceded by *Campylobacter* infection. Intravascular immunoglobulin therapy attenuated the progression of the paralysis.

**Conclusions:**

We report a case of SEA and GBS following *Campylobacter* infection. A combination of the two diseases is rare; however, it could occur if the preceding infection is caused by *Campylobacter* spp. If a cause is known but the patient does not respond to the corresponding treatment, it is important to reconsider the diagnosis based on the medical history.

**Supplementary Information:**

The online version contains supplementary material available at 10.1186/s12883-021-02537-6.

## Background

Spinal epidural abscess (SEA) is generally caused by *Staphylococcus aureus* and *Escherichia coli* and leads to acute flaccid paralysis [1]. Guillain–Barré syndrome (GBS) is an important differential diagnosis of SEA because it also presents with acute flaccid paralysis triggered by an infection [2]. However, the development of GBS following SEA has not been reported.

*Campylobacter jejuni* and *Campylobacter coli* mostly cause *Campylobacter* enteritis. *Campylobacter coli* is closely related to *C. jejuni* in its microbiological and clinical features [3]. *Campylobacter jejuni* is most frequently associated with the onset of GBS [4], but the role of *C. coli* in GBS remains unclear. In addition, SEA due to *Campylobacter* infection is extremely rare, and to the best of our knowledge, there are no reports of SEA caused by *C. coli*.

Here, we report the first case of SEA with GBS following *C. coli* infection.

## Case presentation

**Fig. 1:**
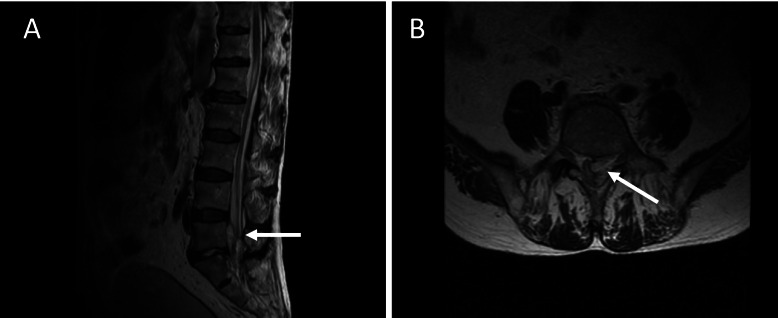
Lumbar magnetic resonance imaging. **a** Sagittal lumber T2-weighted images show a high signal intensity at the L5-S1 vertebral disc, indicating an epidural abscess (arrow). **b** Axial T2-weighted images reveal abscess at the L5 disc (arrow)

A 71-year-old Japanese man presented with lower back pain for 10 days. He was admitted to a local hospital with suspected spondylodiscitis. The patient had a fever and could not move due to severe back pain, but there was no weakness in any of the limbs. He had experienced mild diarrhea 2 weeks before noticing the back pain. His diet often included grilled chicken. The patient’s medical history revealed thoracoplasty for tuberculosis at 25 years of age. After admission, levofloxacin was administered (500 mg/day). Blood culture revealed the presence of *Campylobacter* species. Four days post admission, he experienced left lower limb weakness (Day 1). Magnetic resonance imaging revealed SEA in the spinal region L5-S1 (Fig. 1); therefore, the purulent epidural mass was surgically removed (Day 1). The purulent abscess culture was negative, which we believe is due to the antibiotics that were administered. A day after the surgery, the lower limb paralysis progressed, and then mild weakness appeared bilaterally in the upper limbs (Day 2). Two days after surgery (Day 3), the patient was transferred to our hospital because of the progression of limb weakness. On admission, he was afebrile, and his Glasgow Coma Scale score was 15 (E4V5M6), and his vital signs were unremarkable. Neurological examination revealed proximal and distal bilateral weakness. Deep tendon reflexes were reduced in all limbs, and planter reflexes were indifferent. The senses of touch and vibration were decreased in all distal limbs. There were no signs of cranial nerve abnormality, dermatomal sensory loss, respiratory failure, or urinary retention.

Blood work revealed an elevated white blood cell count of 13,600 cells/μL, a C-reactive protein level of 14.24 mg/dL, and an HbA1c level of 7.2%. Anti-GM1 IgG and IgM, anti-GD1a IgG (ganglioside antibodies), and anti-N-acetylgalactosaminyl GD1a (anti-GalNac-GD1a) IgG were detected. Blood and stool cultures of the respective samples, collected upon admission to our department, were negative. Cerebrospinal fluid analysis was not conducted because the patient was admitted after surgery. There were no abnormalities in the magnetic resonance imaging of the brain and cervix. Lumbar magnetic resonance imaging was performed two and 3 days after the surgery, and there was no progression of SEA. The nerve conduction studies showed conduction blockage in the right ulnar nerve (below elbow-under elbow) upon admission. At 4 days after admission (Day 6), we observed decreased compound muscle and sensory nerve action potentials, with normal right ulnar motor distal latencies but prolonged right median motor distal latency, normal conduction velocities, and absence of F waves, which suggested axonal sensorimotor neuropathy (Supplementary Table 1). The prolonged right median distal latency and absence of F waves were because of distal reversible conduction failure, which was caused by axonal damage at the level of the nerve roots. Therefore, we diagnosed the patient with acute motor sensory axonal neuropathy, which is a variant of GBS. Intravenous immunoglobulin therapy was administered for 5 days at a daily dose of 0.4 g/kg. We could not identify the specific *Campylobacter* spp. The empirical treatment was changed to meropenem (6 g/day), considering the severe infections caused by *Campylobacter* spp. The inflammatory markers improved quickly after surgery. To identify the bacterial species, a strain was isolated from the blood culture performed in the previous hospital, and additional analyses were performed. Polymerase chain reaction (PCR) for *C. coli* was positive. We also confirmed the species as *C. coli*, which lacks the genes *cstIV* and *cstV* that encode sialyltransferases IV and V. Consequently, meropenem was administered for 4 weeks, after which the antibiotic was changed to oral levofloxacin. A glove-sock type of dysesthesia without dermatomal sensory loss gradually became apparent and paralysis of the extremities progressed for 10 days from the onset, and began to improve gradually 14 days after the onset. Five weeks after admission, the patient was transferred to a local hospital for rehabilitation.

## Discussion and conclusions

We describe a case of SEA with acute motor sensory axonal neuropathy (axonal variant of GBS) after *C. coli* infection.

The most common cause of SEA is *Staphylococcus aureus*, followed by gram-negative bacilli, such as *Escherichia coli* [1]. SEA caused by *Campylobacter* infection is extremely rare*. C. jejuni* and *C. coli* infections are major causes of food-borne enteritis in humans. *Campylobacter* enteritis causes *Campylobacter* bacteremia in less than 1% individuals [5]. The incidence of *C. coli* bacteremia remains unknown. To the best of our knowledge, extraintestinal infections due to *Campylobacter* infection, such as SEA caused by *C. jejuni* [6] and spondylitis/spondylodiscitis caused by *C. fetus* and *C. coli* [7], are extremely rare. The pathogenic mechanism of SEA could be attributed to bloodstream infection and direct invasion [1]. Immunosuppressed pediatric and elderly patients are at a risk of bloodstream infections. In the present case, SEA could have been caused by bloodstream infection because the blood culture (at the first hospital) was positive for *C. coli*. Prior administration of levofloxacin might have resulted in the culture being negative for SEA in our hospital. Neurological symptoms of SEA include nervous root symptoms at the level of disability, followed by symptoms related to the spinal cord, including sensory, motor, and bladder-rectal disorders [1]. The distribution of sensory disturbances is associated with the dermatome, and not with the glove and stocking pattern. Cervical SEA can cause quadriplegia that progresses from the upper limbs. In this case, initially paralysis of the lower limbs occurred, which progressed to paralysis of the upper limbs. The dominant distal sensory impairment and hyporeflexia suggested that the paralysis originated from the peripheral nerves rather than from the spinal cord; therefore, the patient was diagnosed to have GBS, which presents with acute flaccid paralysis after infection.

*Campylobacter jejuni* infection is one of the causes of GBS; however, the relationship between *C. coli* infection and GBS remains controversial. *Campylobacter jejuni* has a ganglioside-like lipooligosaccharide (LOS) similar to the gangliosides on human peripheral nerves. This induces anti-ganglioside antibodies, resulting in GBS [8]. Ganglioside is a glycosphingolipid that contains sialic acid in its sugar backbone; therefore, the ganglioside-like LOS must have sialic acid for the *Campylobacter* spp. to induce GBS [8]. *C. jejuni*, which causes GBS, has ganglioside-like LOS with CstII, N-acetylgalactosaminyltransferase, and galactosyltransferase, which are encoded by *cst-II, cgtA*, and *cgtB*, respectively [9]. Furthermore, two novel genes in *C. coli* are possibly involved in the onset of GBS: these are *cstIV* and *cstV*, which induce LOS that contains sialic acid [10]. However, we could not detect these genes in the *C. coli* isolate by PCR. In addition, we could not identify a relationship between *C. coli* infection and GBS in this case. A possible reason for developing GBS could be co-infection. Co-infection of *C. jejuni* and *C. coli* has been identified in 32.1% of chicken liver samples [11]. Furthermore, it has been reported that 3.2% of stool samples from patients with acute flaccid paralysis showed co-infection of *C. jejuni* and *C. coli* [12]. The ganglioside antibodies show a stronger cross-reaction with the LOS of *C. jejuni* than with the LOS of *C. coli* [3]. The types of ganglioside antibodies found in the present case suggested *C. jejuni* infection; however, we could not confirm the infection. It is possible that the stool sample could not be examined during the period of diarrhea, owing to which the co-infection was not detected. Therefore, *Campylobacter* infection, believed to be caused by *C. coli* alone, could be a co-infection of *C. jejuni* and *C. coli*. Therefore, it is necessary to consider that this co-infection may cause GBS.

In conclusion, SEA and GBS can be mimics of each other; however, they can occur simultaneously if they are preceded by *Campylobacter* infection. Therefore, if one pathological condition is diagnosed but does not show a typical response to standard treatment, it is necessary to take into account the medical history of the patient and reconsider the diagnosis.

## Supplementary Information

**Additional file 1: Supplementary Table 1.** Nerve conduction studies (right side). MN: median nerve, UN: ulnar nerve, TN: tibial nerve, SN: sural nerve, MCS: motor conduction study, SCS: sensory conduction study, w: wrist, e: elbow, be: below the elbow, ae: above the elbow, a: ankle, p: popliteal, m: mid-calf, DL: distal latency, CMAP: compound muscle action potential, MCV: motor nerve conduction velocity, F: F-latency, SNAP: sensory nerve action potential, SCV: sensory nerve conduction velocity, NE: not evoked, NP: not performed.

## Data Availability

All data containing relevant information to support the study findings are included in the manuscript.

## Abbreviations

anti-GalNac-GD1a: Anti-N-acetylgalactosaminyl GD1a
GBS: Guillain–Barré syndrome
LOS: Lipo-oligosaccharide
PCR: Polymerase chain reaction
SEA: Spinal epidural abscess
