# Supplementary material for: Campylobacter coli infection causes spinal epidural abscess with Guillain–Barré syndrome: a case report
Source: BMC Neurol. 2022 Jan 3;22:9. doi: 10.1186/s12883-021-02537-6 (PMC8722166; doi:10.1186/s12883-021-02537-6)
Supplement: Supplementary file 1 — Additional file 1: Supplementary Table 1. Nerve conduction studies (right side). MN: median nerve, UN: ulnar nerve, TN: tibial nerve, SN: sural nerve, MCS: motor conduction study, SCS: sensory conduction study, w: wrist, e: elbow, be: below the elbow, ae: above the elbow, a: ankle, p: popliteal, m: mid-calf, DL: distal latency, CMAP: compound muscle action potential, MCV: motor nerve conduction velocity, F: F-latency, SNAP: sensory nerve action potential, SCV: sensory nerve conduction velocity, NE: not evoked, NP: not performed. [file 12883_2021_2537_MOESM1_ESM.docx]

| **Supplementary Table 1. Nerve conduction studies (right).** | | | | | |
| --- | --- | --- | --- | --- | --- |
| Days after the left lower limb weakness onset | 3 | 6 | 10 | 14 | 21 |
| MN-MCS |  |  |  |  |  |
| DL (ms) | 3.5 | 4.3 | 5 | 4.7 | 4.7 |
| CMAP (w/e) (mV) | 5.4/3.9 | 2.5/0.5 | 0.9/0.3 | 0.7/0.3 | 0.7/0.4 |
| MCV (w-e) (m/s) | 52 | 47 | 49 | 50 | 55 |
| F (ms) | 23 | NE | NE | NE | NE |
|  |  |  |  |  |  |
| MN-SCS |  |  |  |  |  |
| SNAP (w) (μV) | 10.2 | 5.9 | 8.5 | 4.5 | NE |
| SCV (w) (m/s) | 56 | 52 | 54 | 53 | NE |
|  |  |  |  |  |  |
| UN-MCS |  |  |  |  |  |
| DL (ms) | 2.2 | 3.1 | 3.4 | NE | NE |
| CMAP (w/be/ae) (mV) | 4.7/4.5/0.1 | 1/0.5/NE | 1/NE/NE | NE | NE |
| MCV (w-be/be-ae) (ms) | 47/37 | 50/NE | NE | NE | NE |
| F (ms) | NE | NE | NE | NE | NE |
|  |  |  |  |  |  |
| UN-SCS |  |  |  |  |  |
| SNAP (w) (μV) | 8.4 | 6.6 | NE | NE | NE |
| SCV (w) (m/s) | 65 | 50 | NE | NE | NE |
|  |  |  |  |  |  |
| TN-MCS |  |  |  |  |  |
| DL (ms) | 6.3 | NP | 4.7 | NE | NE |
| CMAP (a/p) (mV) | 6.2/5.5 | NP | 0.7/NE | NE | NE |
| MCV (a-p) (m/s) | 42 | NP | NE | NE | NE |
| F (ms) | NE | NP | NE | NE | NE |
|  |  |  |  |  |  |
| SN-SCS |  |  |  |  |  |
| SNAP (m) (μV) | 9 | NP | 5.1 | 8.8 | 7.6 |
| SCV (m) (m/s) | 43.5 | NP | 37.6 | 34 | 35 |

MN: median nerve, UN: ulnar nerve, TN: tibial nerve, SN: sural nerve, MCS: motor conduction study, SCS: sensory conduction study, w: wrist, e: elbow, be: below the elbow, ae: above the elbow, a: ankle, p: popliteal, m: mid-calf, DL: distal latency, CMAP: compound muscle action potential, MCV: motor nerve conduction velocity, F: F-latency, SNAP: sensory nerve action potential, SCV: sensory nerve conduction velocity, NE: not evoked, NP: not performed.
